# The Many Faces of Part-List Cuing—Evidence for the Interplay Between Detrimental and Beneficial Mechanisms

**DOI:** 10.3389/fpsyg.2018.00701

**Published:** 2018-05-11

**Authors:** Eva-Maria Lehmer, Karl-Heinz T. Bäuml

**Affiliations:** Department of Experimental Psychology, Regensburg University, Regensburg, Germany

**Keywords:** episodic memory, retrieval cues, forgetting, context reactivation, collaborative inhibition

## Abstract

If participants study a list of items and, at test, receive a random selection of the studied items as retrieval cues, then such cuing often impairs recall of the remaining items. This effect, referred to as part-list cuing impairment, is a well-established finding in memory research that, over the years, has been attributed to quite different cognitive mechanisms. Here, we provide a review of more recent developments in research on part-list cuing. These developments (i) suggest a new view on part-list cuing impairment and a critical role of encoding for the effect, (ii) identify conditions in which part-list cuing impairment can turn into part-list cuing facilitation, and (iii) relate research on part-list cuing to a phenomenon from social memory, known as collaborative inhibition. The recent developments also include a new multi-mechanisms account, which attributes the effects of cuing to the interplay between detrimental mechanisms—like blocking, inhibition, or strategy disruption—and beneficial mechanisms—like context reactivation. The account provides a useful theoretical framework to describe both older and newer findings. It may guide future work on part-list cuing and may also motivate new research on collaborative inhibition.

## Introduction

Recall from episodic memory can benefit greatly from the presence of adequate retrieval cues. For instance, individuals' recall of autobiographical events can improve if information about single aspects of the event—like persons involved or the location of the event—is provided during retrieval (Wagenaar, 1986). People's recall levels are typically higher when retrieval takes place in the same external and internal context as was present during study (Smith and Vela, 2001). Also, people recall more items from a categorized list when they are presented with the category names as a retrieval aid (Tulving and Pearlstone, 1966).

**Figure 1:**
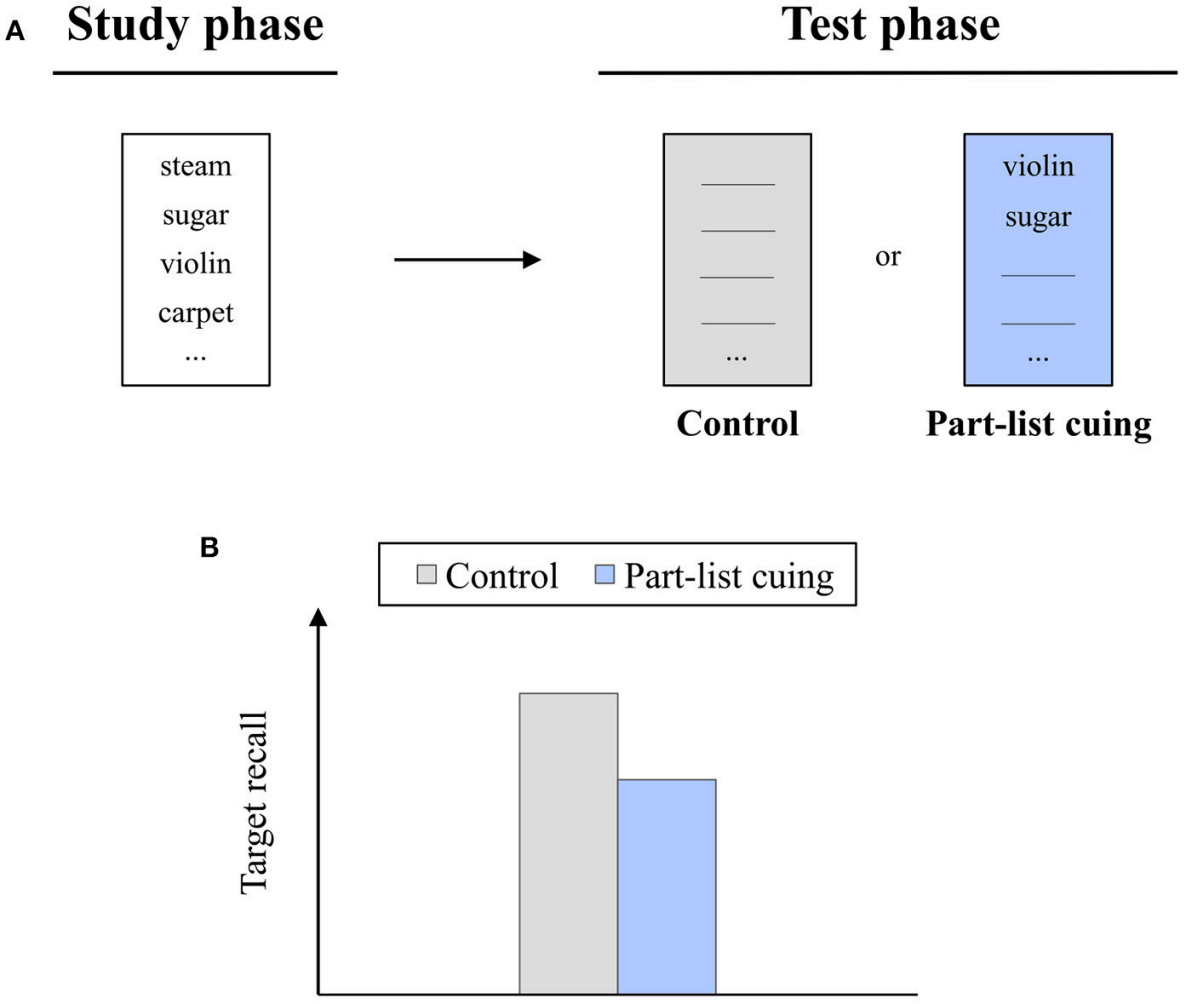
**(A)** The part-list cuing task. Participants study a list of items and, on a later test, are either asked to recall the items in the absence of any retrieval cues (*Control*) or receive a random selection of the studied items as retrieval cues for recall of the remaining (target) items (*Part-list cuing*). **(B)** Typical finding. Recall of the target items is impaired in the part-list cuing relative to the control condition.

However, retrieval cues do not always improve recall performance. For instance, if participants study a list of unrelated items and, at test, receive a random selection of the studied items as retrieval cues, then the beneficial effect of cuing often reverses into a detrimental effect (Slamecka, 1968; Roediger, 1973; see Figure 1). Over the years, numerous studies have reported this detrimental effect, which is referred to as *part-list cuing [PLC] impairment* in the following. PLC impairment has been demonstrated in episodic as well as semantic memory (Brown, 1968; Sloman, 1991), in recognition and reconstruction tasks (Todres and Watkins, 1981; Kelley and Bovee, 2007), and in veridical and false memory settings (Kimball and Bjork, 2002; Bäuml and Kuhbandner, 2003). It has been found with different age groups (Marsh et al., 2004; Zellner and Bäuml, 2005) as well as clinical populations (Bäuml et al., 2002; Christensen et al., 2006).

The present review provides an overview of basic concepts in research on PLC and, in particular, reports on more recent developments in this research area. In the first step, the review will address the question of the cognitive mechanisms mediating PLC impairment and their dependence on encoding. In the second step, the findings from more recent studies will be reviewed, revealing beneficial effects of PLC; possible underlying mechanisms of the facilitation effect will be discussed. Combining the results on the detrimental and beneficial effects of PLC, in the third step, a multi-mechanisms account of PLC will be introduced. Finally, the results from PLC research will be related to a phenomenon from social memory, known as collaborative inhibition, which shows some—empirical and theoretical—parallels to the effects of PLC.

## Accounts of PLC impairment and the role of encoding

### Accounts of PLC impairment

There are three prominent accounts of PLC impairment in the literature: blocking, inhibition, and strategy disruption. The blocking account assumes that the presentation of part-list cues strengthens the cue items' representations, so that, during attempts to recall the remaining (target) items, the stronger cue items are continually brought to mind, blocking access to the target items The inhibition account assumes that the presentation of part-list cues leads to early covert retrieval of the cue items and that this covert retrieval triggers inhibitory processes on the target items, which reduce the activation level of the target items and thus lower chances for the items to be recovered (Anderson et al., 1994; Bäuml and Aslan, 2004). Finally, the strategy disruption account supposes that the presentation of part-list cues at test disrupts retrieval by forcing a serial recall order that is inconsistent with the retrieval plan formed by the participants during encoding (Basden et al., 1977).

Each of the three accounts can deal with a number of findings in the PLC literature, but none of them can explain the whole range of results. For instance, the finding of PLC impairment in item recognition (Todres and Watkins, 1981; Oswald et al., 2006) and forced-order recall tests (Aslan et al., 2007) is consistent with inhibition—which proposes that PLC reduces the targets' representations *per se*—but fits less well with blocking and strategy disruption. In fact, blocking effects are typically absent in item recognition (e.g., Ratcliff et al., 1990; Murnane and Shiffrin, 1991; Rupprecht and Bäuml, 2016), so that, according to the blocking view, no PLC impairment should arise in this type of test, which disagrees with the results of Oswald et al. (2006) and Todres and Watkins (1981). Experimenter-imposed recall orders as induced by item-specific cues should disrupt the learner's retrieval plan, but, according to strategy disruption, should do so irrespective of whether part-list cues are provided or not, which disagrees with the results of Aslan et al. (2007). Also, the finding that PLC impairment can be eliminated in repeated testing situations, in which part-list cues are present on a first recall test but are removed on a second recall test (Basden et al., 1977; Basden and Basden, 1995), is consistent with strategy disruption—which assumes that the disrupted retrieval strategy is quickly reinstated after the removal of the cues. The same finding, however, fits less well with inhibition and blocking, which both assume that the effects of PLC are lasting (for details, see Bäuml and Aslan, 2006). More recent work therefore asked whether a combination of the mechanisms may explain a wider range of PLC findings.

### The role of encoding for PLC impairment

Bäuml and Aslan (2006) suggested a two-factor account of PLC impairment, arguing that both inhibition and strategy disruption contribute to the effect, though in different encoding situations. The authors distinguished between low associative and high associative encoding conditions. In low associative encoding conditions, participants create a relatively low level of interitem associations during study, for instance, by encoding to-be-learned items within a single study cycle without any specific encoding instructions. In contrast, in high associative encoding conditions, participants create a high level of interitem associations, for instance, by receiving repeated study-test cycles (e.g., Tulving, 1962) or the instruction to encode the study items in the presented order (e.g., Basden et al., 2002). The main idea of the account then is that the degree of interitem associations influences which cognitive mechanism is triggered. A low degree of interitem associations may cause a high amount of interitem interference, inducing inhibition when part-list cues are provided; in contrast, a high degree of interitem associations may result in a preferred output order that can easily be disrupted by the presentation of a random set of studied items serving as part-list cues.

**Figure 2:**
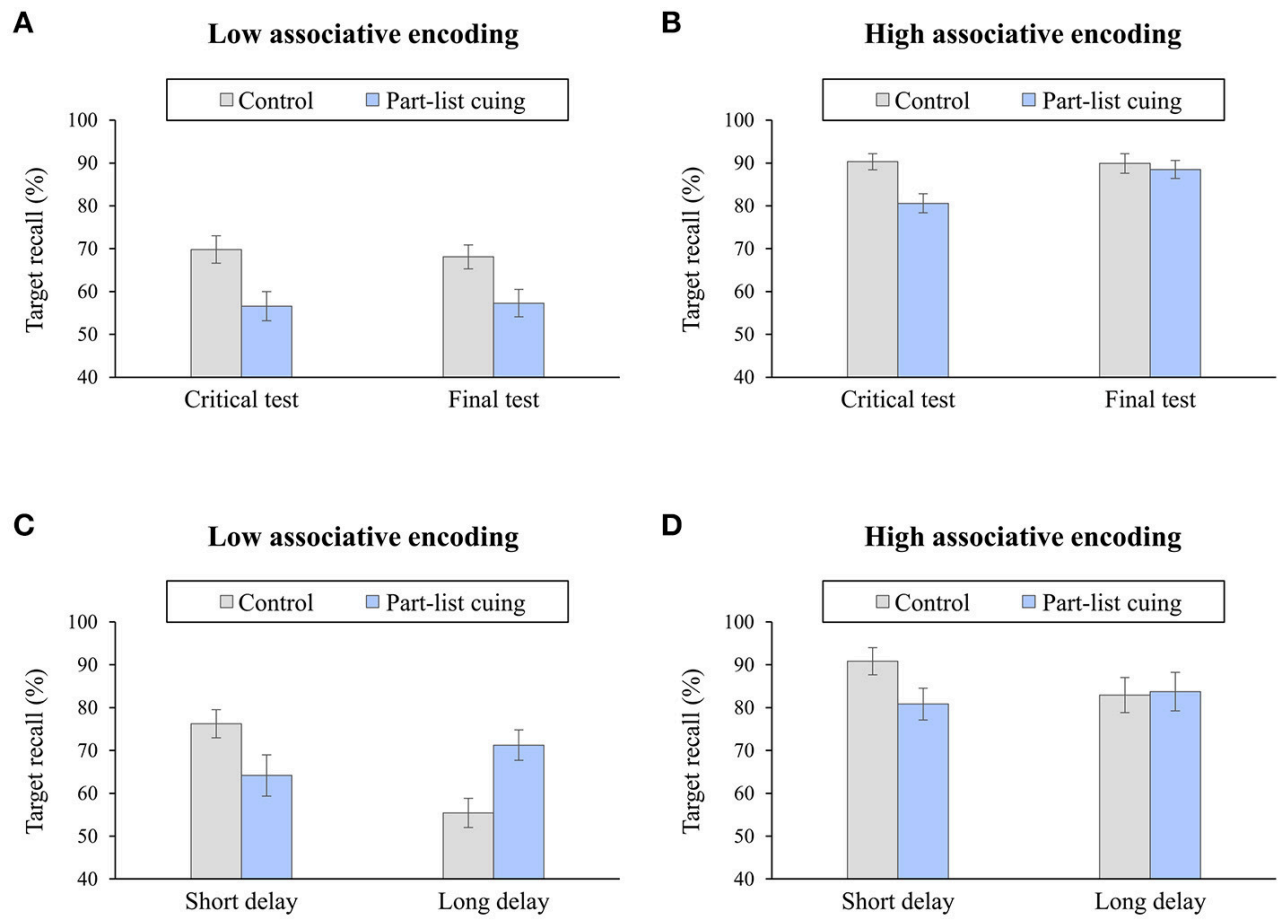
**(A,B)** Part-list cuing effects after short delay as a function of encoding (low associative, high associative) and test (critical, final). Recall results are shown for a repeated testing situation, in which part-list cues are present on a first, critical test, but are removed on a second, final test. Part-list cuing impairment persists on the second test with low associative encoding, but disappears on the second test with high associative encoding. Adapted from Bäuml and Aslan (2006). **(C,D)** Part-list cuing effects as a function of encoding (low associative, high associative) and delay (short, long). With low associative encoding, part-list cuing impairment is present after short delay, whereas part-list cuing facilitation arises when the retention interval is prolonged. With high associative encoding, part-list cuing impairment is present after short delay, but recall is unaffected by the cues after prolonged retention interval. Adapted from Lehmer and Bäuml (2018).

The two-factor account is consistent with many findings in PLC research (for a summary, see Bäuml and Aslan, 2006), but Bäuml and Aslan also tested the account more directly, investigating detrimental effects of PLC in different encoding situations. In the first step, Bäuml and Aslan (2006) examined whether the effects of repeated testing—in which part-list cues are provided on a first recall test, but are removed on the second test—depend on encoding; in the second step, Aslan and Bäuml (2007) examined whether the presence of unique initial-letter cues, serving as item-specific cues for the target items, influences the effects of PLC, and whether this influence varies with encoding. Encoding influenced the results in both studies: Whereas, with repeated testing, PLC impairment disappeared after the removal of the cues with high associative encoding, it persisted with low associative encoding (see Figures 2A,B; but see Muntean and Kimball, 2012); when item-specific probes were provided at test, PLC impairment was present with low associative encoding, but it was absent with high associative encoding. These findings fit with the two-factor account and the view that inhibition primarily operates with low associative encoding and strategy disruption primarily operates with high associative encoding.

## PLC facilitation

### Beneficial effects of PLC

While traditionally PLC research has focused almost exclusively on detrimental effects of PLC (for exceptions, see Serra and Nairne, 2000; Basden et al., 2002), the results from three studies demonstrate that, under certain circumstances, PLC can also improve recall performance. All of these studies employed low associative encoding. In the first study, Goernert and Larson (1994) examined the effects of PLC in listwise directed forgetting. In this task, participants study a list of items and then, after study, are asked either to continue remembering or to forget the list. After learning of another list, the first-list items are tested, and participants typically recall fewer items in the forget than in the remember condition (Bjork, 1970). Goernert and Larson (1994) employed this task but additionally varied whether, during first-list recall, part-list cues were provided or not. The results showed typical PLC impairment in the remember condition, but PLC facilitation in the forget condition.

Bäuml and Samenieh (2012) replicated the finding and generalized it to context-dependent forgetting. Subjects studied two lists of items and, between study of the two lists, completed a neutral counting task or changed their internal context by means of an imagination task (see Sahakyan and Kelley, 2002). After study of the second list, participants recalled first-list items, in either the presence or the absence of part-list cues. PLC impaired target recall after the counting task but improved target recall after the imagination task. An analogous pattern arose in time-dependent forgetting, when recall was tested after either a delay of a few minutes or a prolonged retention interval of 48 h. PLC impairment arose when testing occurred after short delay, but PLC facilitation was found when the retention interval was prolonged (Bäuml and Schlichting, 2014).

### Mechanisms of PLC facilitation and the role of encoding

Because after a forget cue, a context-change task, or a prolonged retention interval access to study context is typically impaired at test (e.g., Estes, 1955; Geiselman et al., 1983; Sahakyan and Kelley, 2002), the finding of PLC facilitation in the three studies may point to a role of context reactivation in PLC. Consistently, Bäuml and Samenieh (2012) reasoned that, when access to study context is impaired, PLC may trigger processes that reactivate the original study context, which may then serve as a retrieval cue for the target items and improve recall performance. This proposal is consistent with research in other areas, like the spacing effect (e.g., Greene, 1989) or the contiguity effect (e.g., Howard and Kahana, 1999), in which selective item repetition, be it via restudy or retrieval, has also been suggested to induce context reactivation.

While the prior work thus suggests that context reactivation can induce beneficial effects of PLC with low associative encoding, this work leaves it open whether there is a similar role of context reactivation with high associative encoding. Lehmer and Bäuml (2018) addressed the issue, employing both low and high associative encoding to examine possible beneficial effects of PLC. Access to study context at test was manipulated using listwise directed forgetting as well as short vs. prolonged retention intervals. With low associative encoding, the previously observed pattern of detrimental and beneficial effects of PLC was replicated, with detrimental effects of PLC when access to study context at test was maintained and beneficial effects when the access was impaired. With high associative encoding, again detrimental effects of PLC arose when study context access was maintained, but PLC left recall unaffected when the access was impaired (see Figures 2C,D). Beneficial effects of PLC arose on the second test of a repeated-testing task only, when part-list cues were provided on the first recall test but were removed on the second test. These results suggest a role of context reactivation for PLC also with high associative encoding, although, with this type of encoding, beneficial effects on recall may be masked by strategy disruption processes as long as the part-list cues are present (see below).

## A multi-mechanisms account of PLC

On the basis of the recent findings, Lehmer and Bäuml (2018) suggested a multi-mechanisms account of PLC. The basic assumption of this account is that, in general, PLC triggers both detrimental mechanisms (inhibition, blocking, strategy disruption) and beneficial mechanisms (context reactivation). The detrimental mechanisms operate primarily when at test the access to the study context is maintained, whereas the beneficial mechanism operates primarily when the access is impaired. Besides, it is assumed that different detrimental mechanisms are triggered in different encoding conditions: inhibition and blocking with low associative and strategy disruption with high associative encoding. Context reactivation is supposed to operate independently of encoding and benefit recall by reinstating the original encoding context.

These assumptions lead to the expectation that, when at test the access to the study context is maintained, PLC will impair recall because the relative contribution of the detrimental mechanisms will be larger than of context reactivation. When at test the access to the study context is impaired, however, the relative contribution of context reactivation should increase and PLC no longer impair recall. Whether, under such conditions, PLC induces beneficial or neutral effects on recall should depend on encoding. With low associative encoding, context reactivation should improve recall because the relative contribution of context reactivation is supposed to be larger than of inhibition and blocking (see Bäuml and Samenieh, 2012). With high associative encoding, however, the relative contributions of context reactivation and strategy disruption can be more similar. Indeed, as argued by Lehmer and Bäuml, under these conditions, the context reactivation can lead to relatively fast reconstruction of the original retrieval plan, so that the potentially beneficial effect of context reactivation as caused by the part-list cues can be masked by the detrimental effect of strategy disruption caused by the same part-list cues, which, as a net result, can leave recall unaffected. Importantly, in a repeated-testing situation, in which part-list cues are present on a first recall test but are removed on the second, the masking effect should be eliminated on the second test and the beneficial effects of context reactivation become manifest. The results of Lehmer and Bäuml (2018) reported above show exactly such a pattern and thus support the proposal.

## Application to social memory

### Collaborative inhibition and suggested mechanisms

In the past two decades, a growing number of studies investigated the effects of collaboration on memory performance and linked the observed recall impairment in collaborative groups to the detrimental effects of PLC. In a typical collaborative memory experiment, participants individually study materials and, after a short retention interval, are asked to recall the studied items, either individually or in a collaborating group. Recall performance of the collaborating group is then compared with recall performance of a so-called nominal group. A nominal group consists of an equal number of participants working individually and recall performance is calculated by pooling their non-redundant answers. While collaborative groups recall more than their individual members, collaborative groups recall less than nominal groups, a finding termed collaborative inhibition (Weldon and Bellinger, 1997; Rajaram and Pereira-Pasarin, 2010).

Collaborative inhibition is a robust and fairly general finding that arises with a variety of study materials, like word lists, prose texts, or pictures (Weldon and Bellinger, 1997; Finlay et al., 2000). Following the strategy disruption account of PLC impairment, the leading theoretical explanation is that participants develop their individual retrieval strategies during encoding and that, at test, these strategies are disrupted by the outputs of the other group members (Basden et al., 1997). The disruption hypothesis is supported by studies that demonstrate a release of collaborative inhibition on a subsequent individual recall task (Basden et al., 1997; Finlay et al., 2000), and it is challenged by studies that demonstrate collaborative inhibition in forced-order tests, like cued recall or item recognition (Andersson and Rönnberg, 1996; Kelley et al., 2012). Alternative accounts also follow PLC research and suggest a role of blocking and inhibition in this form of recall impairment (for a meta-analysis, see Marion and Thorley, 2016), although no clear suggestions have yet been made about when the single mechanisms should operate. Future work may examine whether encoding plays a similar role in collaborative inhibition as it plays in PLC impairment, which would help to better understand whether similar mechanisms contribute to the two forms of recall impairment.

### Collaborative recall and context reactivation

More recently, Abel and Bäuml (2017) investigated if, analogous to PLC, access to study context at test can influence the effects of collaboration. Participants studied a list of unrelated words and were tested either individually or in collaborating groups. Additionally, access to study context at test was manipulated by employing a remember or a forget instruction after study, and by varying the length of the retention interval between study and test. Typical detrimental effects of collaboration arose when access to study context was maintained, i.e., after a remember instruction or a short retention interval, but these effects were eliminated once study context access was impaired, i.e., after the forget instruction or a prolonged retention interval (see also Takahashi and Saito, 2004; Congleton and Rajaram, 2011).

One reason for why collaborative inhibition disappears after a forget cue or prolonged delay may be that the role of strategy disruption or inhibition is reduced when study context access is impaired, be it because subjects no longer rely as heavily on their idiosyncratic retrieval strategies or because the interference level of the items is reduced (see Takahashi and Saito, 2004). Alternatively, the finding may reflect the action of context reactivation processes. Here the proposal is that when the overlap between study and test context is high, primarily strategy disruption or inhibition may operate and induce recall impairment, whereas when the overlap between study and test contexts is reduced, context reactivation may play a more important role and at least eliminate the impairment effect. Interestingly, the results by Abel and Bäuml (2017) parallel the PLC effects observed by Lehmer and Bäuml (2018) with high associative encoding, which is consistent with the view that strategy disruption contributes to recall in social settings and both strategy disruption and context reactivation operate when access to study context at test is impaired. Further studies are required to investigate the possible role of context reactivation in collaborative recall in more detail.

## Conclusions

PLC has many faces. It can impair, improve, or not affect recall performance, depending on type of encoding and access to study context at test. PLC typically impairs recall if at test access to study context is maintained, but this detrimental effect can turn into a neutral or even beneficial effect if at test access to study context is impaired. A multi-mechanisms account of PLC, which attributes the effects of PLC to the interplay between detrimental and beneficial mechanisms, provides a useful theoretical framework to describe the existing results and may help to guide future work on PLC. Such future work may also include studies on social memory and the effects of collaboration on recall performance.

## Author contributions

All authors listed have made a substantial, direct and intellectual contribution to the work, and approved it for publication.

### Conflict of interest statement

The authors declare that the research was conducted in the absence of any commercial or financial relationships that could be construed as a potential conflict of interest.
